# Comparative Field Evaluation and Transcriptome Analysis Reveals that Chromosome Doubling Enhances Sheath Blight Resistance in Rice

**DOI:** 10.1186/s12284-024-00722-y

**Published:** 2024-07-03

**Authors:** Sanglin Liu, Jiahao Liu, Wei Wang, Yugang Yan, Tianya Wang, Jinwen Wu, Xiangdong Liu, Jian Wu, Yuxiang Zeng

**Affiliations:** 1 State Key Laboratory for Conservation and Utilization of Subtropical Agro-Bioresources; Guangdong Laboratory for Lingnan Modern Agriculture, South China Agricultural University, Guangzhou, 510642 China; 2 Guangdong Provincial Key Laboratory of Utilization and Conservation of Food and Medicinal Resources in Northern Region, School of Biology and Agriculture, Shaoguan University, Shaoguan, 512005 China; 3 State Key Laboratory of Rice Biology and Breeding, China National Rice Research Institute, Hangzhou, 310006 China; 4 Guangdong Provincial Key Laboratory of Plant Molecular Breeding, College of Agriculture, South China Agricultural University, Guangzhou, 510642 China

**Keywords:** Rice, Autotetraploid, Sheath blight resistance, Transcriptome

## Abstract

Rice sheath blight, caused by *Rhizoctonia solani* Kihn (*R. solani*), poses a significant threat to rice production and quality. Autotetraploid rice, developed through chromosome doubling of diploid rice, holds great potential for enhancing biological and yield traits. However, its resistance to sheath blight in the field has remained unclear. In this study, the field resistance of 35 autotetraploid genotypes and corresponding diploids was evaluated across three environments from 2020 to 2021. The booting stage was optimal for inoculating period based on the inoculation and analysis of *R. solani* at five rice growth stages. We found autotetraploids generally exhibited lower disease scores than diploids, indicating enhanced resistance after chromosome doubling. Among the 35 genotypes, 16 (45.71%) displayed increased resistance, 2 (5.71%) showed decreased resistance, and 17 (48.57%) displayed unstable resistance in different sowing dates. All combinations of the genotype, environment and ploidy, including the genotype-environment-ploidy interaction, contributed significantly to field resistance. Chromosome doubling increased sheath blight resistance in most genotypes, but was also dependent on the genotype-environment interaction. To elucidate the enhanced resistance mechanism, RNA-seq revealed autotetraploid recruited more down-regulated differentially expressed genes (DEGs), additionally, more resistance-related DEGs, were down-regulated at 24 h post inoculation in autotetraploid versus diploid. The ubiquinone/terpenoid quinone and diterpenoid biosynthesis pathways may play key roles in ploidy-specific resistance mechanisms. In summary, our findings shed light on the understanding of sheath blight resistance mechanisms in autotetraploid rice.

**Supplementary Information:**

The online version contains supplementary material available at 10.1186/s12284-024-00722-y.

## Background

Rice sheath blight, caused by *Rhizoctonia solani* Kihn (*R. solani*), has a widespread impact on rice production worldwide (Li et al. 2021; Molla et al. 2020). It can reduce rice yields by up to 50% and dramatically affect rice quality, threatening global food security (Abdelsalam et al. 2020; Singh et al. 2019). The disease is difficult to control due to lack of resistant rice cultivars and favorable conditions for *R. solani*, such as warm temperatures and high humidity (Kouzai et al. 2018). Moreover, *R. solani* has a broad host range and survive for long time in soil as sclerotia, climate change may exacerbate sheath blight by creating more suitable conditions for the pathogen (Kamboj et al. 2016; Taheri et al. 2007).

Infected rice leaves by sheath blight exhibit grayish-brown, cloud-like lesions and eventually wilt (Lee 1983; Li et al. 2020a). Currently, commercial fungicides are the main preventive measure, which lead to environmental pollution. Breeding resistant rice varieties is also challenging, due to the lack of resistant germplasms and quantitative resistance genes confer small effects (Li et al. 2021; Zhao et al. 2008). Many rice accessions from different countries have been screened for sheath blight resistance (Jia et al. 2012; Srinivasachary et al. 2011; Taguchi-Shiobara et al. 2013; Yadav et al. 2015), but none were completely immune, only showing reduced symptoms in varieties with better resistance (Srinivasachary et al. 2011; Zeng et al. 2017). However, varieties and lines with partial resistance offer potential for breeding sheath blight resistant cultivars under field conditions. Sato et al. (2004) used a resistant line whose resistance was derived from Tetep. Yangdao 4, a cultivar with partial resistance to ShB, has been reported to be the most resistant cultivar among 68 cultivars inoculated with the *R. solani* isolate RH-9 (Pan et al. 2001). Promising sheath blight resistance has been identified in cultivars including Teqing (Li et al. 1995), Jasmine85 (Park et al. 2008), Tetep (Channamallikarjuna et al. 2010), Jarjan (Taguchi-Shiobara et al. 2013), Yangdao 4 (Zeng et al. 2015) and ARC10531 (Yadav et al. 2015), providing resources for resistant variety breeding and offering hope for disease control (Zeng et al. 2015).

The lack of effective genetic resistance to sheath blight in commercial rice has driven efforts to develop resistant lines via breeding and transgenics. RNA-seq has provided insights into resistance genes and rice-pathogen interactions for sheath blight (Rao et al. 2020; Yang et al. 2022). Identification of differentially expressed genes (DEGs) following *R. solani* infection by RNA-seq has elucidated rice defense mechanisms to some extent. Multiple transcriptomic studies have elucidated mechanisms of *R. solani* pathogenesis and rice sheath blight resistance. Xia et al. (2017) and Rao et al. (2019) analyzed *R. solani* isolates from different hosts, identifying polygalacturonase (PG) as a key virulence factor. These transcriptomic studies have revealed new aspects of *R. solani* pathogenicity and host resistance. Zhang et al. (2017) compared RNA-seq profiles of the moderately resistant Teqing and susceptible Lemont after *R. solani* infection, finding jasmonic acid (JA) signaling, phenylpropanoid metabolism, photosynthesis and photorespiration contribute to enhanced resistance observed in Teqing. RNA-seq analysis of the resistant Shennong 9819 and susceptible Koshihikari showed faster defense pathway activation and up-regulation of pathogenesis-related (PR) genes, transcription factors, and phenylalanine ammonia lyase (PAL) genes in the resistant line during early *R. solani* infection (Yang et al. 2022). Other comparative transcriptomic studies with various sheath blight tolerant and susceptible rice genotypes identified DEGs patterns underlying resistance, using CR 1014 (Samal et al. 2022), ZhengDao 22 (Yang et al. 2023), GD66 (Liu et al. 2022), and YSBR1 (Zheng et al. 2018) as the tolerant genotypes. In summary, comparative rice transcriptomics has revealed key pathways and DEGs involved in sheath blight infection and defense.

Autotetraploid rice is a novel germplasm obtained from diploid rice by chromosomes doubling (Wu et al. 2021). Autotetraploids have many advantages over diploids, including stronger stems, larger/heavier grains, and higher protein and amino acid content (Tu et al. 2007; Wu et al. 2013, 2014). However, the impact of chromosome doubling on field resistance to various rice diseases is still unknown. This study utilized 35 autotetraploid rice genotypes and corresponding diploids, previously developed and characterized for genetic diversity and embryo sac fertility by our group (Hu et al. 2009; Wu et al. 2013). The field resistance of different ploidy rice to sheath blight was evaluated across three environments in Guangzhou from 2020 to 2021. Three environments referred to distinct planting seasons, with materials being planted on March 01, 2020, July 26, 2020, and February 25, 2021. Autotetraploids exhibited lower disease scores and higher resistance than diploids, but the effect varied between genotypes and environments. Genotype, environment, ploidy and their interactions significantly influenced field resistance. The RNA-seq analysis comparing autotetraploid and diploid genotypes revealed more up-regulated gene induction at 24 h post inoculation (hpi) in autotetraploids, potentially explaining enhanced resistance. Further analysis revealed that genes involved in the ubiquinone/terpenoid quinone and diterpenoid biosynthesis pathways may have significant roles in the sheath blight disease resistance of autotetraploid rice. These findings have important implications for elucidating disease resistance mechanisms in autotetraploid rice.

## Materials and Methods

### Rice Genotype and Growing Environment

The present study included 35 rice autotetraploid genotypes, which were derived from corresponding diploids by colchicine-induced chromosome doubling as described previously (Hu et al. 2009; Wu et al. 2013). The 35 autotetraploid rice genotypes and corresponding diploids are listed in Additional file 1: Table S1. Additionally, Lemont (E240) and Yinhesizhan (E266) were obtained from the Chinese National Rice Research Institute (CNRRI), the resistance of them was described previously (Zeng et al. 2017). All the genotypes were grown in a greenhouse with natural light and 20–35 °C or in the field with standard practices at South China Agricultural University (23.16 N, 113.35 E), Guangzhou, China. To evaluate field resistance to sheath blight, the 35 autotetraploids and 35 diploids were planted during three seasons at the experimental farm of South China Agricultural University in Guangzhou from 2020 to 2021: (1) March 01, 2020 (spring of 2020); (2) July 26, 2020 (summer of 2020); (3) February 25, 2021 (a replication of spring in 2021 compared to the spring of 2020). Each genotype was planted in a plot of 50 plants, arranged in 5 rows of 10 plants with 10 cm spacing between rows and plants. The plots were randomly distributed to different locations.

### Inoculation and Evaluation of Sheath Blight Field Resistance

The rice plants were inoculated with the *R. solani* isolate ZJ03 as described previously (Zeng et al. 2017; Zou et al. 2000) with minor modifications: ZJ03 was incubated on potato dextrose agar (PDA) medium (200 g of potato, 20 g of dextrose, and 20 g of agar for 1 L) for 3 days in darkness at 28 °C. Truncated bamboo toothpicks (2–2.5 cm) penetrated the 5 mm diameter PDA medium covered with mycelia were inserted into the third leaf sheath from the top at booting stage. At this growth stage, the second sheath was no longer growing, therefore the toothpick remained stable inside the third sheath (Xue et al. 2016).

The sheath blight field resistance was evaluated by recording lesion length (LL, cm), disease rating (DR), and 7-rating score (7R). The lesion length was measured from the lowest to the highest point of the lesion along the stem at 10 and 28 days post inoculation (dpi). Five plants in the middle row were inoculated for each genotype at booting stage with two tillers per plant, resulting in 10 inoculated tillers per genotype. The lesion length was recorded at 10 and 28 dpi. Disease rating followed the 0–9 rating system (Xu 2016), where 0 indicates a plant immune to sheath blight, 9 indicates a plant completely affected by the disease leading to death, and 5 indicates that about 50% of the plant was affected by the disease (see Additional file 1: Table S2). The 7-rating score (7R) was used to assess the overall field resistance of a genotype in each planting environment (the materials were planted on March 1, 2020, July 26, 2020, and February 25, 2021). Based on the overall field resistance, the resistance levels were subjectively evaluated and categorized into four distinct levels: highly resistant (HR), moderately resistant (MR), moderately susceptible (MS), and highly susceptible (HS). The 7-rating scores were as follows: 1 for HR, 3 for MR, 5 for MS, and 7 for HS. All statistical analyses, including two-way and three-way ANOVA, were performed in RStudio. The correlation coefficient was calculated using R, and the results were displayed using the ‘corrplot’ library in RStudio.

### Transcriptome Analysis

The susceptible diploid cultivar Bengal (E29) and its resistant autotetraploid Bengal-4X (T49, see Additional file 1: Table S1) were used for transcriptome analysis by RNA-seq. Young seedlings were greenhouse-grown then transferred to a growth chamber (DHP-9052, Shanghai, China, 28 °C, 16 h light/8 h dark) at booting stage for 10 days pre-inoculation. The toothpicks penetrating the 5 mm diameter PDA medium covered with mycelia were used for sheath inoculation, following the method described by Zeng et al. (2017). Total RNA was extracted from inoculated autotetraploid and diploid sheathes at 24 and 48 hpi, with blank medium inoculated as the control. Sheath tissue near the inoculation site was collected in liquid nitrogen and stored at − 80 °C. RNA extraction, library construction and Illumina HiSeqTM2500 sequencing were performed by Biomarker Technologies (Beijing, China) (Yu et al. 2018). Differentially expressed genes (DEGs) were identified in DESeq with fold change (FC) ≥ 2 and false discovery rate (FDR) ≤ 0.01. GSEA (Gene Set Enrichment Analysis), KEGG and GO enrichment of DEGs were conducted using BMKCloud (https://www.biocloud.net). Cluster analysis was performed in TBtools to generate heatmaps (Chen et al. 2020).

### qRT-PCR Analysis of Candidate Genes

Eleven candidate genes were selected for qRT-PCR validation using gene-specific primers designed in Primer Premier 5.0 (Additional file 1: Table S3). RNA was extracted using Trizol reagent (Invitrogen, Waltham, MA, USA) and reverse transcribed with the Evo M-MLV RT Kit (Accurate Biotechnology, Hunan, China) according to the manufacturer’s protocols. qRT-PCR was performed on the Lightcycler480 (Roche, Basel, Switzerland) using the Hieff qPCR SYBR Green Master Mix (Yeasen, Shanghai, China). The 20 μL reactions were conducted as follows: 95 °C for 30 s, followed by 45 cycles of 95 °C denaturation for 10 s, and 58 °C annealing and extension for 20 s. Relative expression was calculated by the 2^−ΔΔCt^ method (Livak and Schmittgen 2013), with rice *ubiquitin* as the internal control (Wu et al. 2014). Three replicates were used for all samples.

## Results

### Assessing the Optimal Inoculation Stage for Rice Genotypes with Field Sheath Blight Resistance

**Fig. 1:**
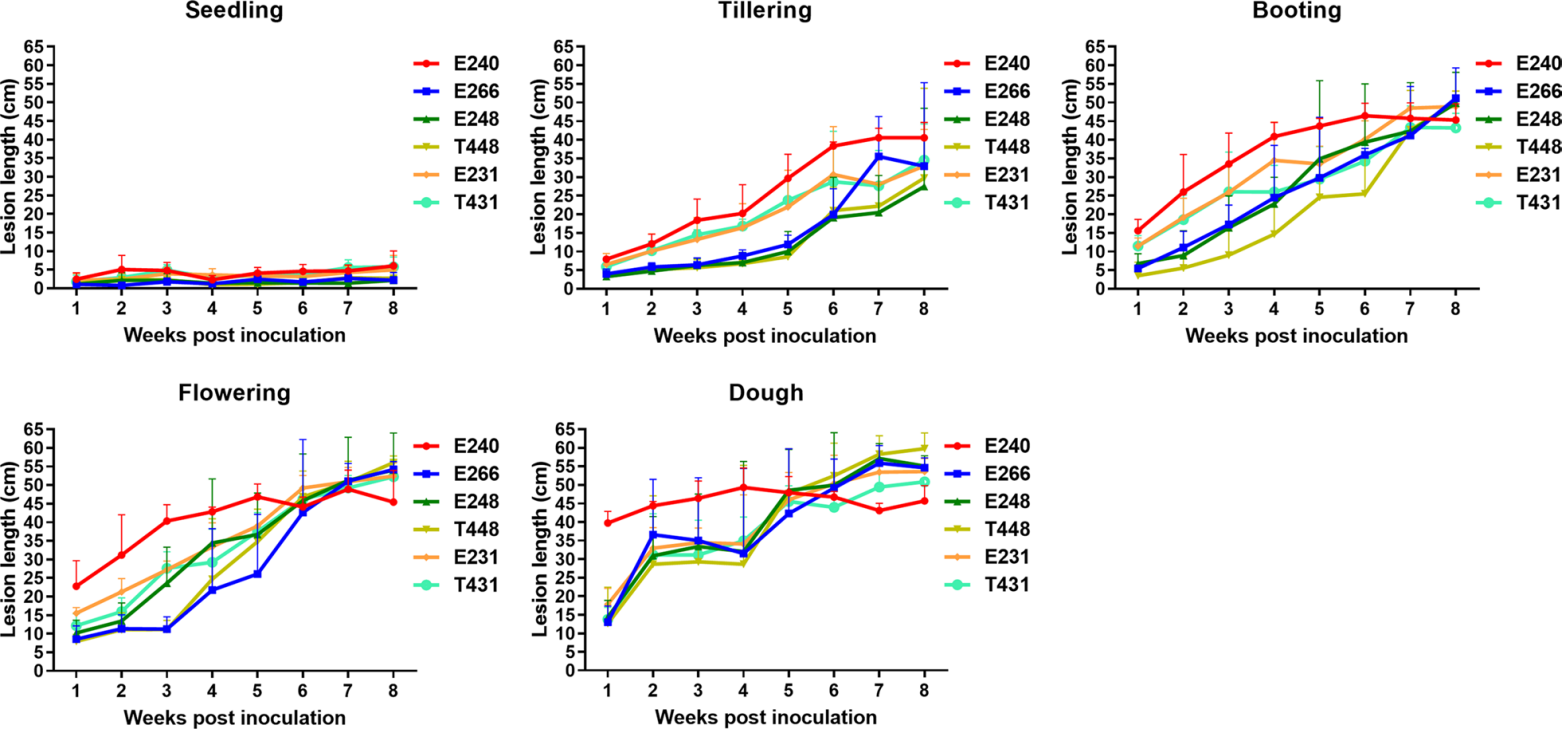
The lesion length of sheath blight at different growth stages post inoculation

To identify the optimal inoculation stage, six rice genotypes with different field resistances to sheath blight (Table 1) were inoculated at several growth stages, including seedling, tillering, booting, flowering and dough stages. The lesion length on the 6 genotypes was recorded weekly for a total of eight times after inoculation. The assessment was repeated 3 times. Our results revealed that the disease severity was lowest at seedling stage and progressed more rapidly at later stages (Fig. 1).

**Table 1:** Phenotype of the genotypes in the field. Lesion length and disease rating were recorded at 28 dpi

Genotype	Code	Average lesion length (cm)	Alpha = 0.05	Average disease rating	Alpha = 0.05	Subjective evaluation^①^
8821	E248	36.1 ± 4.6	a	4.3 ± 0.4	a	HR
Taichung65-4x	T431	38.6 ± 2.1	a	4.3 ± 0.4	a	MR
Yinhesizhan	E266	42.4 ± 5.2	a	5.1 ± 0.6	ab	MR
8821-4x	T448	36.9 ± 5.8	a	5.3 ± 0.7	b	MR
Taichung65	E231	50.0 ± 6.6	b	6.0 ± 0.9	b	MS
Lemont	E240	59.4 ± 2.2	c	7.5 ± 0.3	c	HS

^①^HS, highly susceptible; MS, moderately susceptible; MR, moderately resistant; HR, highly resistant

The analysis of variance (ANOVA) revealed that the three experimental variations, namely stage (i.e. inoculation at different growth stage), evaluation for disease resistance at different duration of weeks post-inoculation (i.e. statistical week), and genotype, had an extremely significant effect on lesion length (Table 2). No significant differences existed between genotypes inoculated at the seedling stage. However, seven, six and three pairs of genotypes differed significantly when inoculated at the tillering, booting, and flowering stages, respectively. Only E240 and T431 were significantly different when inoculated at the dough stage (Additional file 2: Fig. S1). These results suggest that the effect of genotype on lesion length depends on the inoculation stage, and that some genotypes are more resistant than others at certain stages. There were no significant differences between the lesion lengths recorded at 8 statistical weeks after inoculation at the seedling stage. However, 17, 21, 21 and 19 pairs of statistical weeks’ data differed significantly when inoculated at tillering, booting, flowering and dough stages, respectively (Additional file 2: Fig. S1). These results indicate significant interactions between the three experimental variations on lesion length. In contrast, inoculations at tillering or booting stages caused significant differences between resistant and susceptible genotypes. Similarly, inoculations at booting or flowering resulted in significant differences between statistical weeks. Based on these results, we concluded that the booting stage was the optimal inoculation stage, and we selected it as the primary inoculation stage for further analysis.

**Table 2:** Analysis of variance (ANOVA) for lesion length in response to rice sheath blight in different genotypes, growth stages, and statistical weeks (i.e. evaluation for disease resistance at different durations of weeks post-inoculation)

Origin of variation	Type III square	df	Mean square	F value	*P*
Stage	126,954.6	4	31,738.7	501.2	< 0.001
Statistical week	64,695.4	7	9242.2	145.9	< 0.001
Genotype	6889.5	5	1377.9	21.8	< 0.001
Stage × statistical week	19,586.2	28	699.5	11.1	< 0.001
Stage × genotype	2797.1	20	139.9	2.2	0.002
Statistical week × genotype	3675.4	35	105.0	1.7	0.012
Stage × statistical week × genotype	6017.2	140	43.0	0.7	0.997

### Lesion Length Observed at 10 dpi and 28 dpi

**Fig. 2:**
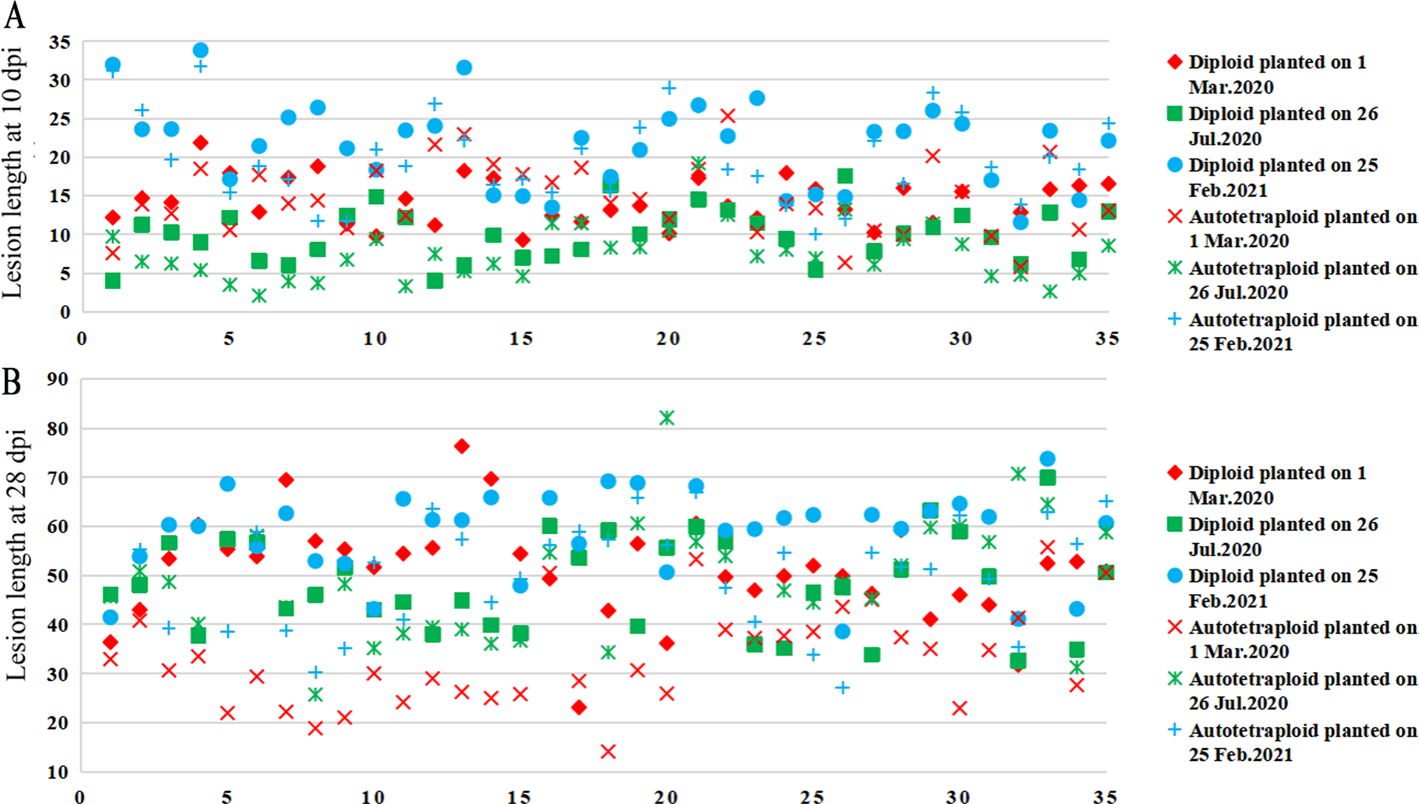
The field resistance of 35 diploid and autotetraploid rice genotypes was measured at 10 dpi and 28 dpi across three environments, which were planted on March 01, 2020, July 26, 2020, and February 25, 2021, respectively. **a** Lesion length measured at 10 dpi. **b** Lesion length measured at 28 dpi. The numbers on the horizontal axis indicate the 35 genotypes. Refer to Additional file 1: Table S1 for the names of the 35 genotypes

Lesion length at 10 dpi and that at 28 dpi were compared using 35 diploid rice varieties and their corresponding autotetraploid counterparts across three different sowing dates (Fig. 2a, b). The correlation coefficients for lesion lengths measured at 10 dpi and 28 dpi ranged from 0.01 to 0.62 in autotetraploids (Additional file 1: Table S4) and from 0.15 to 0.56 in diploids (Additional file 1: Table S5). Additionally, the correlation between lesion length and disease rating (or the 7 rating system score) observed was low at 10 dpi (Additional file 1: Table S4 and S5). In contrast, the correlation coefficients between lesion length and disease rating (or the 7 rating system score) at 28 dpi were higher than those at 10 dpi in most cases (Additional file 1: Table S4 and S5). This indicated that 28 dpi was better than 10 dpi for evaluating field sheath blight resistance in rice.

### Genotype by Environment Interaction Significantly Affects Sheath Blight Field Resistance

**Fig. 3:**
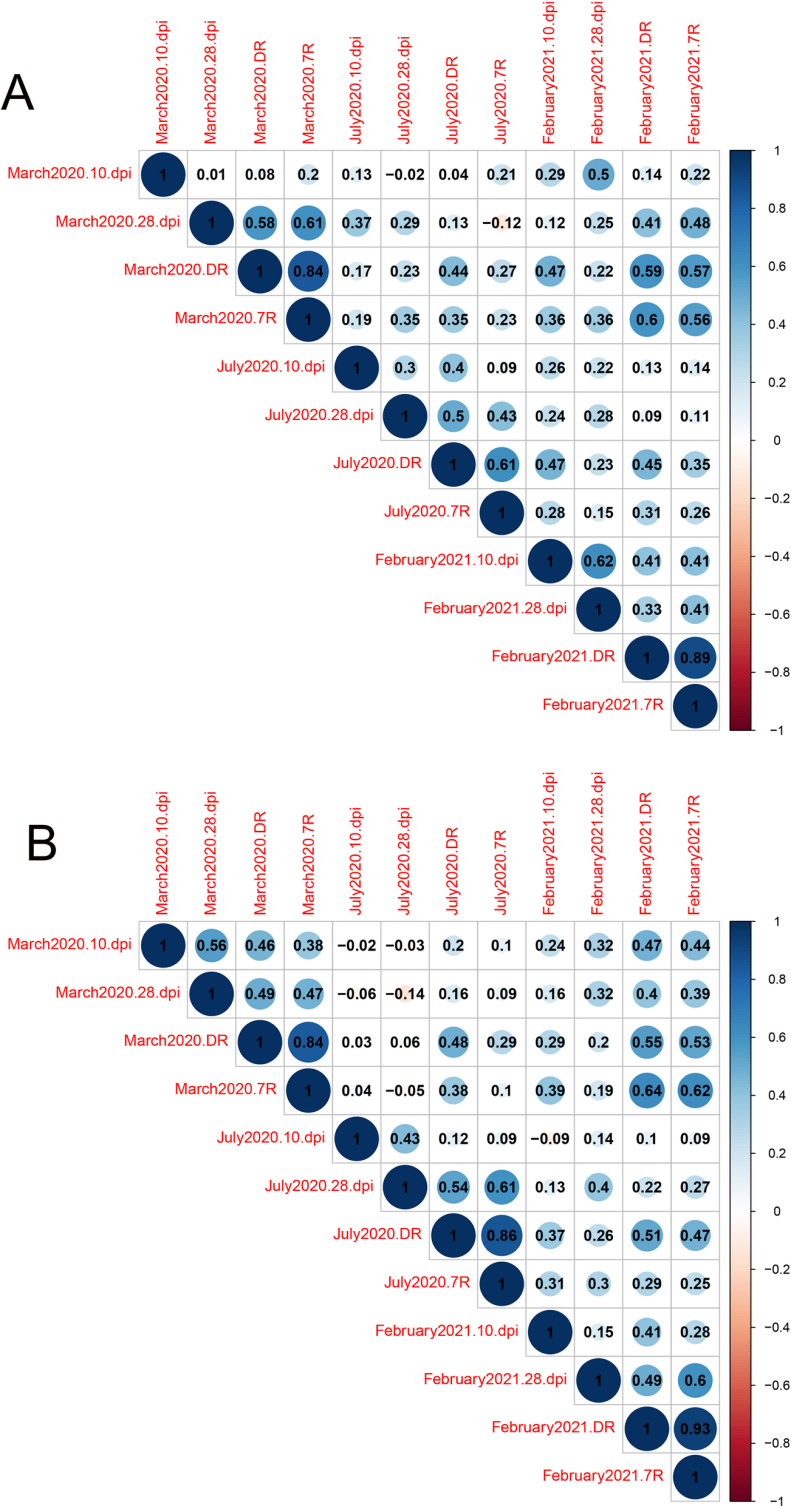
Heatmap of the correlation coefficients for sheath blight field resistance among 35 autotetraploid genotypes (**a**) and corresponding diploid genotypes (**b**) across three environments (materials were planted on March 01, 2020, July 26, 2020, and February 25, 2021). Correlation coefficients were calculated among environments. 10 pi, lesion length at 10 days post inoculation; 28 pi, lesion length at 28 days post inoculation; DR, disease rating; 7R, the 7 rating system

To explore the impact of genotype by environment interaction on the field resistance to sheath blight in 35 diploid and their corresponding autotetraploid rice genotypes, four traits were measured: (1) lesion length at 10 dpi, (2) lesion length at 28 dpi, (3) disease rating using the 0–9 rating system, and (4) the 7 rating system score. The genotypes were grown in three different sowing dates (genotypes were planted on March 01, 2020, July 26, 2020, and February 25, 2021), resulting in 12 sub-traits (4 traits × 3 environments). We computed the correlation coefficients among the 12 sub-traits for the 35 autotetraploid genotypes (Fig. 3a) and 35 diploid genotypes (Fig. 3b), respectively, and displayed them in a heat map generated using the ‘pheatmap’ library in RStudio. Out of the 66 correlation coefficients among the 12 sub-traits, only 12 (18%) were above 0.5, while 54 (82%) were below 0.5 for both autotetraploids (Fig. 3a) and diploids (Fig. 3b), suggesting that the field resistance to sheath blight was not stable across different sowing dates.

Two-way analysis of variance was performed to examine how genotype and environment affect the field resistance to sheath blight in 35 autotetraploid and 35 diploid rice genotypes. It showed that all three variations (genotype, environment, and genotype by environment interaction) had a significant impact on the sheath blight field resistance of both autotetraploid and diploid genotypes (Additional file 1: Table S6–S8). For both autotetraploids and diploids, lesion length at 10 dpi was influenced more by the environment than by the genotype or the genotype by environment interaction, while the genotype itself contributed the least to the total variance (Additional file 1: Table S6). These results confirmed the unsuitability of 10 dpi for field sheath blight evaluation. For autotetraploids, lesion length at 28 dpi was affected by the environment, genotype, and genotype by environment interaction in a similar proportion (35%, 33.74%, and 31.26% of the total variance, respectively), with the environment having a slightly higher contribution than the genotype (Additional file 1: Table S7). For both autotetraploids and diploids, disease rating was determined more by the genotype than by the environment or the genotype by environment interaction (Additional file 1: Table S8).

### Multiple Factor Interaction Significantly Influences Sheath Blight Field Resistance

A three-way analysis of variance was performed to examine the influence of genotype, environment, and ploidy on sheath blight field resistance. All three variations were highly significant for sheath blight resistance (*P* < 0.0001). The two-way interactions (genotype by environment, genotype by ploidy, and environment by ploidy) and three-way interaction (genotype by environment by ploidy interaction) had a highly significant influence on the lesion length and disease rating (*P* < 0.001) (Additional file 1: Table S9–S11). The environment accounted for the most variance of the lesion length at 10 dpi (55.29%), followed by genotype by environment interaction (18.64%), genotype (12.70%), and genotype by environment by ploidy interaction (6.16%) (Additional file 1: Table S9). The genotype by environment interaction accounted for the most variance of the lesion length at 28 dpi (21.45%), followed by genotype (17.49%), environment (16.18%), and genotype by ploidy interaction (14.78%), while the genotype by environment by ploidy interaction accounted for 10.39% of the variance (Additional file 1: Table S10). For disease rating, genotype accounted for the most variance (44.36%), followed by genotype by environment interaction (20.28%), genotype by ploidy interaction (16.05%), and genotype by environment by ploidy interaction (9.14%) (Additional file 1: Table S11). The three-way analysis of variance showed that ploidy and its interactions with genotype and environment had a significant effect on the field resistance to sheath blight when comparing autotetraploids with their corresponding diploids.

### Chromosome Doubling Enhances Sheath Blight Resistance in Autotetraploid Rice Compared to Diploid Rice

**Fig. 4:**
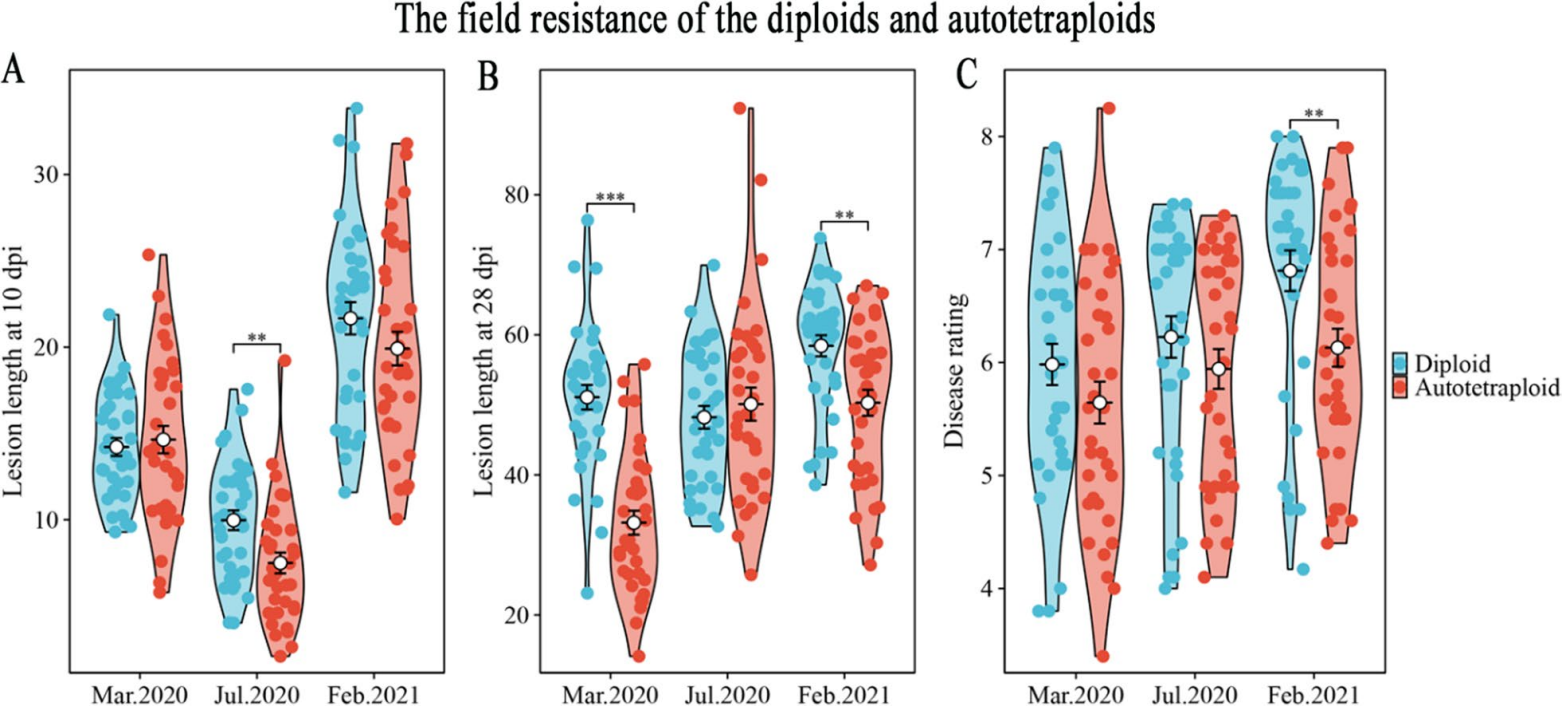
The lesion length and disease rating of 35 diploid and autotetraploid rice genotypes were measured in the field at 10 and 28 dpi across three environments (materials were planted on March 01, 2020, July 26, 2020, and February 25, 2021). **a** Lesion length of the rice genotypes measured at 10 dpi. **b** Lesion length of the rice genotypes measured at 28 dpi. **c** Disease rating of the rice genotypes measured at 28 dpi

The 35 autotetraploids, derived from their corresponding diploids by chromosome doubling, provide a good opportunity to study the effect of chromosome doubling on sheath blight field resistance. We compared the average lesion length (observed at both 10 dpi and 28 dpi), average disease rating, and average 7 rating score across three environments (materials were planted on March 01, 2020, July 26, 2020, and February 25, 2021) between autotetraploid and diploid genotypes. These four traits were used across three environments to assess the impact of chromosome doubling on sheath blight resistance. The original sheath blight field resistance data for autotetraploid and diploid genotypes are provided in Additional file 1: Table S12 and S13, respectively. After chromosome doubling, 16 (45.71%), 2 (5.71%), and 17 (48.57%) showed higher, lower, and unstable sheath blight resistance, respectively, across three environments. These 16 autotetraploid genotypes with higher resistance were L202-4x, 96,025-4x, Jackson-4x, Liaojing944-4x, Yanjing48-4x, Bengal-4x, Jingxian89-4x, J455-4x, Nanhaizaoyinzhan-4x, Guinongzhan-4x, Taichung65-4x, E4-4x, E24-4x, E45-4x, 4001-4x, and IR36-4x. The 2 autotetraploid genotypes with lower resistance were Dayebai-4 × and Goulianzao-4x. Detailed information about these autotetraploid genotypes can be found in Additional file 1: Table S1. It is worth noting that the sheath blight resistance phenotypic data of autotetraploid rice were overall better than that of diploids, indicating enhanced resistance in autotetraploid rice overall compared to diploids (Fig. 4a, b, c and Additional file 1: Table S14).

Due to unstable sheath blight disease scores observed across three sowing dates for certain genotypes, we conducted an analysis of average sheath blight resistance of the 35 genotypes in three sowing dates. This analysis involved four traits: lesion length at 10 days post-inoculation, lesion length at 28 days post-inoculation, disease rating, and a 7-rating score, across the three sowing dates. Based on the average sheath blight resistance data, we found that 24 (68.6%) of the autotetraploid genotypes exhibited enhanced disease resistance compared to their diploid counterparts. Additionally, 7 (20%) of them demonstrated lower disease resistance, while 4 (11.4%) showed nearly equal resistance to their diploid counterparts (Additional file 1: Table S15). These results suggest that chromosome doubling could enhance sheath blight field resistance in most diploid rice varieties. The average values for the four resistance traits were combined and calculated, which showed that three of the most resistant genotypes were Yanjing48-4x, Jingxian89-4x, Bengal-4 × for autotetraploids; and 8821-2x, Huajingxian74-2x, Dayebai-2 × for diploids (Additional file 1: Table S16). Furthermore, we recorded plant height of the autotetraploid and diploid genotypes in both 2020 and 2021 (Additional file 1: Table S17 and S18), and used correlation analysis to explore the relationship between plant height and sheath blight field resistance. We did not find a significant correlation between plant height and lesion length. Significant negative correlations (*p* < 0.01) between plant height and disease rating were detected (Additional file 1: Table S19).

### Transcriptome Analysis of Autotetraploid and Diploid

To analyze the transcriptional response to *R. solani* infection in different ploidy rice, RNA sequencing was performed on the susceptible diploid cultivar Bengal (E29) and its resistant autotetraploid T49 (derived from diploid Bengal by chromosome doubling) at 0, 24, and 48 hpi, with triplicate biological replicates. A total of 127.63 Gb of clean data were obtained, with Q_30_ bases > 94.15%. Alignment to the Nipponbare reference genome ranged from 81.05% to 92.73% (Additional file 1: Table S20).

**Fig. 5:**
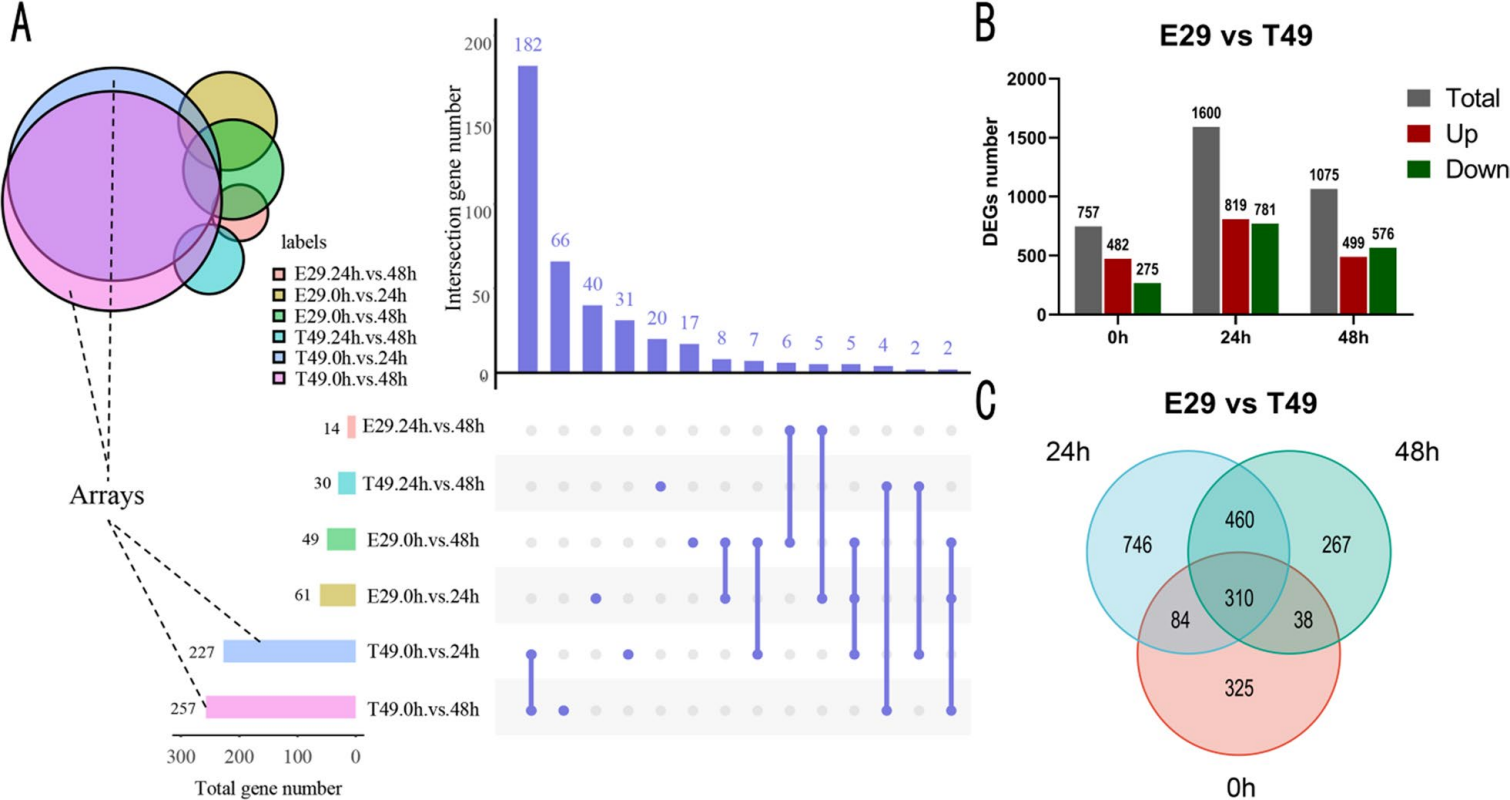
Number of the DEGs in comparisons between three time points for the genotypes*.* **a** UpSet plot showing total and overlapping DEGs across E29 and T49 comparisons. Colored circles indicate DEGs in Venn diagram with matching colored bars, left x-axis denotes DEG counts; right x-axis displays comparison-specific (dots) and shared (connected dots) DEGs. **b** Number of up-regulated (red) and down-regulated (green) DEGs between E29 and T49 at three time comparisons. **c** Venn diagram of overlapping DEGs at three time points

At 24 h post-inoculation (hpi), we detected 61 differentially expressed genes (DEGs) in the susceptible E29. Among them, 24 genes were significantly up-regulated, and 37 genes were significantly down-regulated (E29-0 hpi vs E29-24 hpi) (Table 3, Fig. 5a). In the resistant T49, we observed a total of 227 DEGs at 24 hpi, with 28 genes significantly up-regulated and 199 genes significantly down-regulated (T49-0 hpi vs T49-24 hpi) (Table 3, Fig. 5a). These findings suggest that sheath blight infection leads to more down-regulated genes in autotetraploid than in diploid plants at 24 hpi.

**Table 3:** Number of DEGs after inoculation with *R. solani* in E29 and T49

Comparison	Total DEGs	Up-regulated	Down-regulated
E29-0 hpi vs E29-24 hpi	61	24	37
T49-0 hpi vs T49-24 hpi	227	28	199
E29-0 hpi vs E29-48 hpi	49	39	10
T49-0 hpi vs T49-48 hpi	257	37	220
E29-24 hpi vs E29-48 hpi	14	12	2
T49-24 hpi vs T49-48 hpi	30	9	21
E29-0 hpi vs T49-0 hpi	757	482	275
E29-24 hpi vs T49-24 hpi	1600	819	781
E29-48 hpi vs T49-48 hpi	1075	499	576

At 48 hpi, in the susceptible E29, we identified 39 significantly up-regulated genes and 10 significantly down-regulated genes (E29-0 hpi vs E29-48 hpi). However, in the resistant T49, we found 37 significantly up-regulated genes and 220 significantly down-regulated genes at 48 hpi (T49-0 hpi vs T49-48 hpi) (Table 3). These results indicate that sheath blight infection causes more down-regulated genes in autotetraploid than in diploid at 48 hpi.

We conducted an analysis of down-regulated genes between autotetraploid and diploid plants at 24 hpi. Our findings revealed that more genes involved in the ‘plant-pathogen interaction (ko04626)’ and ‘MAPK signaling pathway (ko04016)’ KEGG pathways were down-regulated in autotetraploid genotype compared to diploid genotype. In the ‘plant-pathogen interaction (ko04626)’ pathway, two genes (*LOC_Os02g12420*, *LOC_Os05g51190*) were significantly down-regulated in E29 (E29-0 hpi vs E29-24 hpi), whereas nine genes (*LOC_Os01g02750*, *LOC_Os04g02120*, *LOC_Os07g26430*, *LOC_Os11g37759*, *LOC_Os11g37774*, *LOC_Os11g45130*, *LOC_Os11g45840*, *LOC_Os12g11500*, *LOC_Os12g30070*) were significantly down-regulated in T49 (T49-0 hpi vs T49-24 hpi). In the ‘MAPK signaling pathway (ko04016)’, only one gene (*LOC_Os09g18159*) was significantly down-regulated in E29 (E29-0 hpi vs E29-24 hpi), while five genes (*LOC_Os09g19229*, *LOC_Os09g19380*, *LOC_Os09g19390*, *LOC_Os09g19400*, *LOC_Os11g45920*) were significantly down-regulated in T49 (T49-0 hpi vs T49-24 hpi). Additionally, we found that five genes (*LOC_Os02g02660*, *LOC_Os11g47290*, *LOC_Os11g47300*, *LOC_Os11g47310*, *LOC_Os12g41560*) in the ‘Plant hormone signal transduction (ko04075)’ pathway were significantly down-regulated in T49 at 24 hpi, whereas none of the genes in the ko04075 pathways were down-regulated in E29 (Additional file 1: Table S21 and S22).

We also compared the down-regulated genes between autotetraploid and diploid plants at 48 hpi. In the ‘plant-pathogen interaction’ (ko04626) pathway, ten genes (*LOC_Os01g02750*, *LOC_Os01g02770*, *LOC_Os04g02120*, *LOC_Os07g26430*, *LOC_Os08g10430*, *LOC_Os11g37759*, *LOC_Os11g37774*, *LOC_Os11g45130*, *LOC_Os11g45840*, *LOC_Os12g30070*) were significantly down-regulated in T49, while no genes were down-regulated in E29. Regarding the ‘MAPK signaling pathway (ko04016)’, six genes (*LOC_Os09g19229*, *LOC_Os09g19350*, *LOC_Os09g19380*, *LOC_Os09g19390*, *LOC_Os09g19400*, *LOC_Os11g45920*) were significantly down-regulated in T49, while no genes were down-regulated in E29. Furthermore, in the ‘Plant hormone signal transduction (ko04075)’ pathway, four genes (*LOC_Os02g02660*, *LOC_Os11g47290*, *LOC_Os11g47300*, *LOC_Os12g41560*) were significantly down-regulated in T49, but no genes in the same pathway were down-regulated in E29 (Additional file 1: Table S23 and S24). These results suggest that the increased sheath blight resistance in T49 may be attributed to a higher number of down-regulated genes in these pathways.

### KEGG Pathway Analysis of the DEGs

**Fig. 6:**
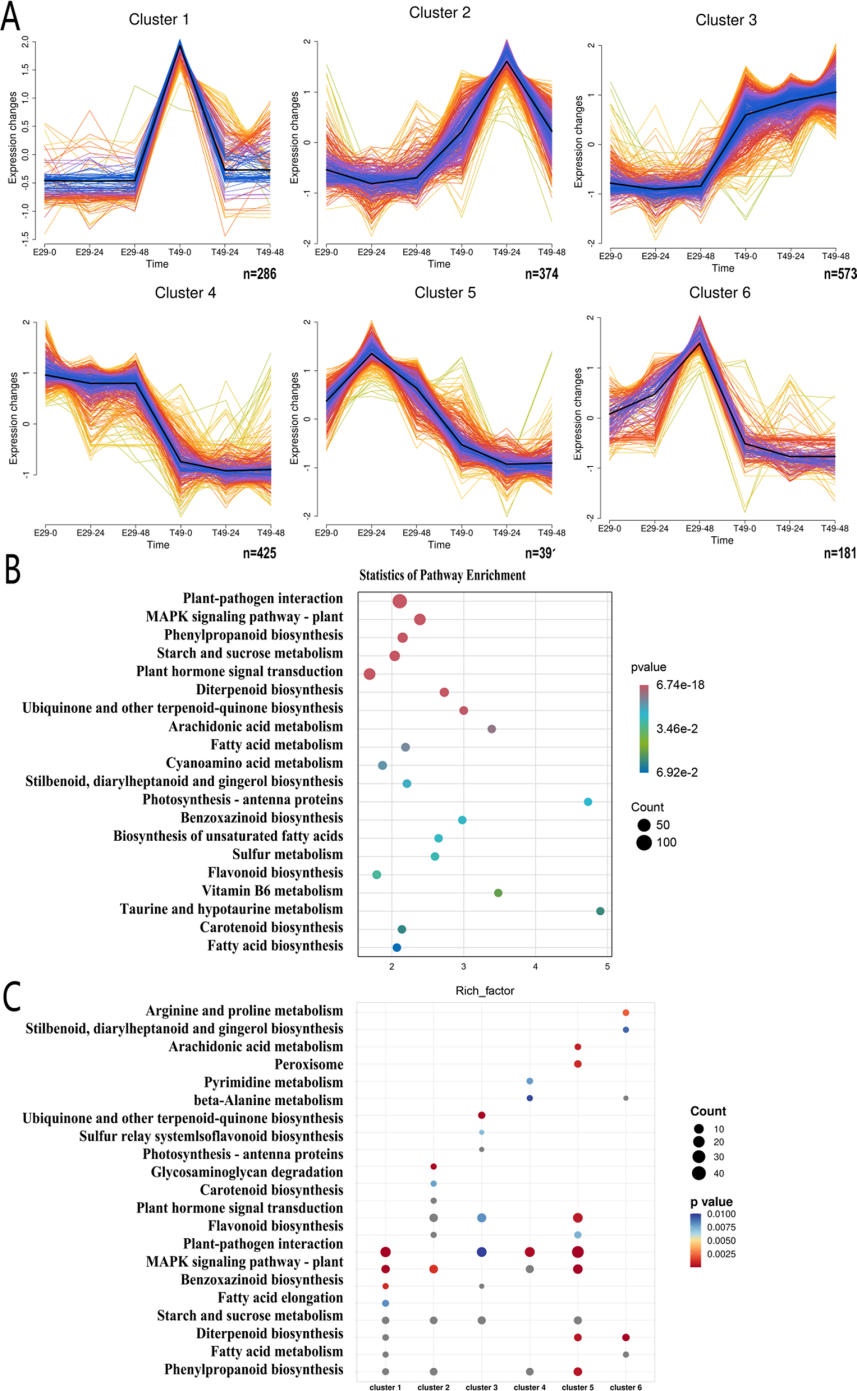
Cluster and KEGG pathway analysis of the DEGs between E29 and T49. **a** Cluster analysis identified 6 distinct temporal expression profiles of DEGs using Mfuzz, the x-axis represents inoculation time points, y-axis represents the FPKM normalized intensity ratio of gene expression at each time. **b** KEGG pathway enrichment analysis of the total DEGs. **c** Visualization of enriched KEGG pathways for DEGs in the 6 clusters

We compared the differentially expressed genes (DEGs) between E29 and T49 at three time points: E29-0 hpi vs T49-0 hpi, E29-24 hpi vs T49-24 hpi, and E29-48 hpi vs T49-48 hpi (Fig. 5b, c, Table 3). This comparison resulted in a total of 2230 DEGs (Fig. 5c). To further investigate the patterns of gene expression induced by *R. solani* infection in susceptible E29 and resistant T49 at different time points, we clustered these 2230 DEGs into six clusters (Fig. 6a). Among these clusters, cluster 3 had the highest number of DEGs (573), while DEGs in clusters 2 were activated at 24 or 48 hpi in T49 compared to E29. These genes may positively regulate resistance to *R. solani*. On the other hand, DEGs in clusters 5 and 6 were up-regulated in E29 after infection (Fig. 6a). Furthermore, KEGG enrichment analysis of the 2230 DEGs identified 115 significantly enriched pathways, including plant-pathogen interaction (KO04626), plant hormone signal transduction (KO04075), MAPK signaling (KO04016), starch/sucrose metabolism (KO00500), phenylpropanoid biosynthesis (KO00940), diterpene biosynthesis (KO00904), and ubiquinone and other terpenoid quinone biosynthesis (KO00130) (Additional file 1: Table S25, Fig. 6b). Genes in clusters 5 and 6 exhibited high expression in E29 at 24 and 48 hpi, respectively, and were enriched in the KO00904 pathway (Fig. 6c).

**Fig. 7:**
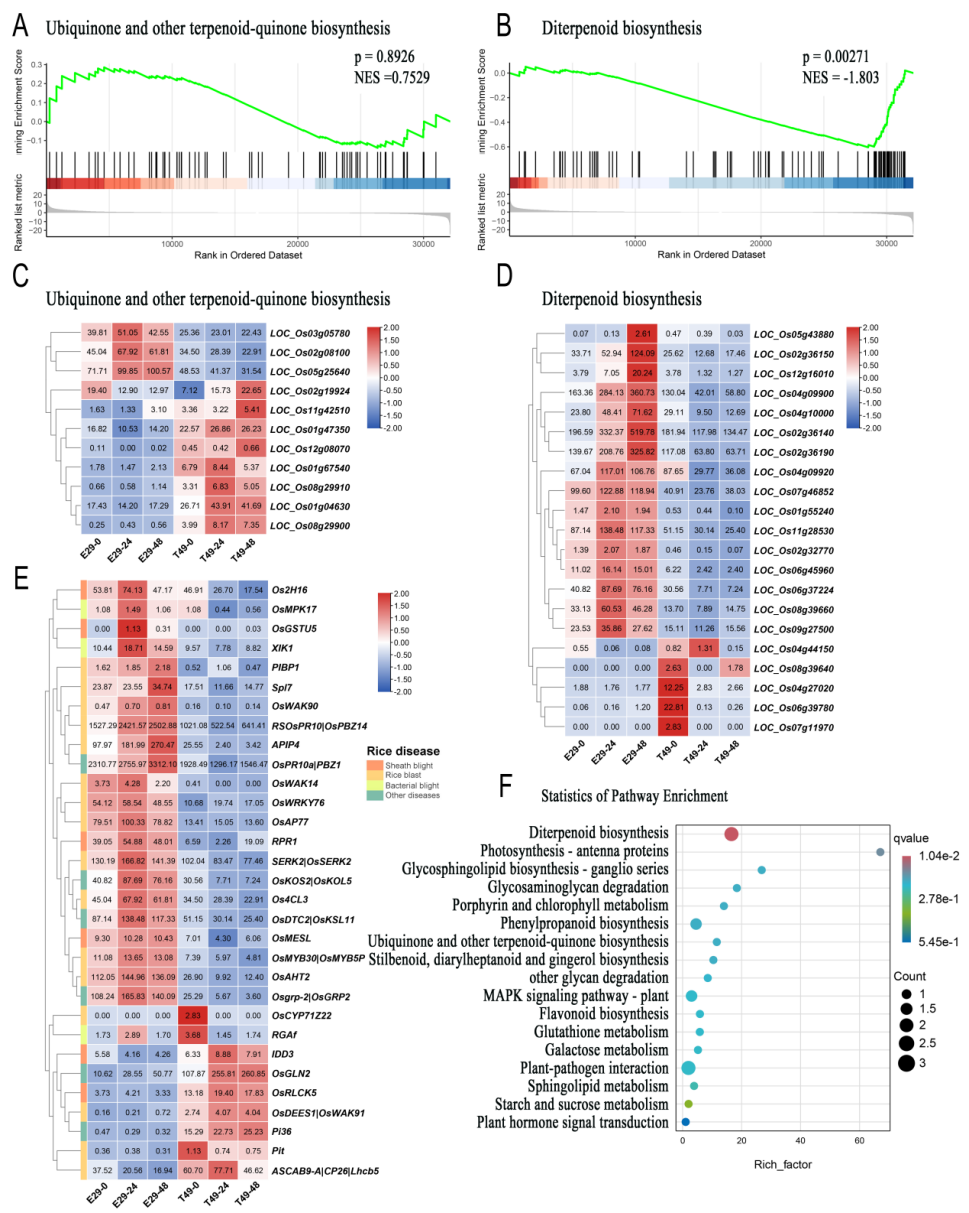
*R. solani* infection affects signaling regulatory pathways in different ploidy rice. GSEA showing **a** DEGs in T49 positively correlated with ubiquinone and other terpenoid quinone biosynthesis, **b** DEGs negatively correlated with diterpenoid biosynthesis. **c**–**d** Detailed expression profiles of genes involved in ubiquinone and other terpenoid quinone and diterpenoid biosynthesis pathways. **e** Expression patterns of 31 disease resistance genes in E29 and T49, values represent FPKM of genes. **f** KEGG analysis of the disease resistance genes

To further explore the KO00904 and KO00130 pathway, we performed gene set enrichment analysis (GSEA). We use the GEAS result to evaluate whether most genes in this pathway were up-regulated or down-regulated. If the Normalized Enrichment Score (NES) was larger than 0 in the GSEA analysis, it suggested that most genes in this pathway were up-regulated. Conversely, if the NES value was lower than 0, it indicated that most genes in this pathway were down-regulated. The GSEA results demonstrated that the majority of the DEGs in the KO00130 pathway were up-regulated in T49 (Fig. 7a, c), whereas most DEGs in the KO00904 pathway were down-regulated (Fig. 7b, d). This finding may potentially explains the enhanced resistance in autotetraploid rice. The related KEGG maps are shown in Additional file 2: Fig. S2 and S3, respectively. Detailed information was listed in Additional file 1: Table S26 and S27.

We examined the 2230 DEGs detected between E29 and T49 (E29-0 hpi vs T49-0 hpi, E29-24 hpi vs T49-24 hpi, and E29-48 hpi vs T49-48 hp), We found that 31 of these DEGs were disease resistance genes previously reported in studies. These 31 genes are listed in Fig. 7e. These genes have been reported to participate in resistance against various diseases, such as sheath blight, rice blast, and bacterial blight. Interestingly, we observed that the majority of these 31 disease resistance genes were down-regulated in the resistant T49 (Fig. 7e). Further analysis revealed that these genes were significantly enriched in the diterpenoid biosynthesis (KO00904) pathway (Fig. 7f), indicating a potential role for negative regulators within diterpenoid biosynthesis in plant defense mechanisms.

### Validation of RNA-seq Results Using qRT-PCR

**Fig. 8:**
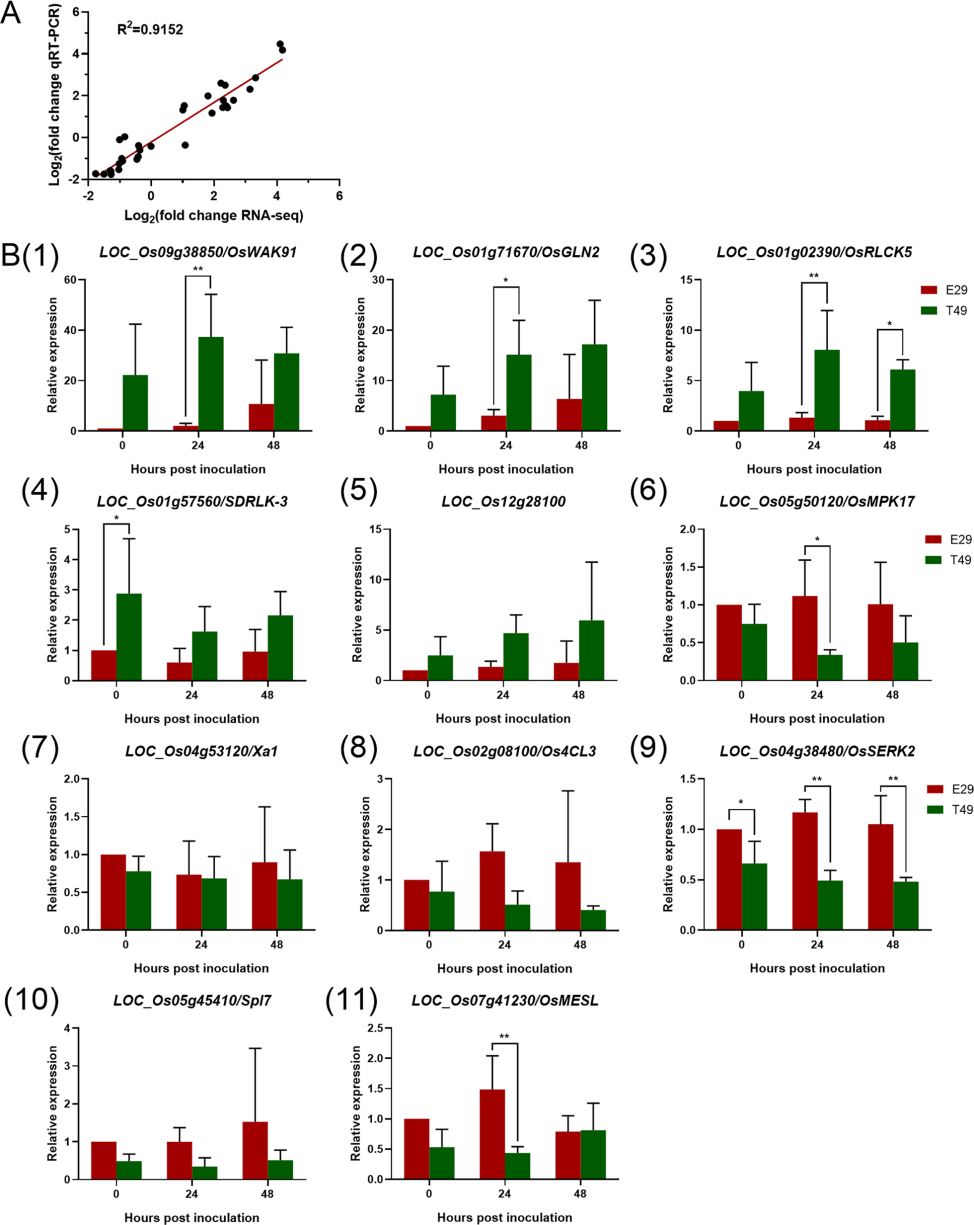
qRT-PCR verification of the RNA-seq results. Eleven DEGs identified in this study were verified using qRT-PCR. Ubiquitin was used as the internal qRT-PCR reference. Error bars signify the standard deviation (SD) across three biological replicates. **a** There was a strong correlation between the qRT-PCR and RNA-seq results. **b** qRT-PCR analysis of 11 genes in both E29 and T49. The 11 genes have been reported to be associated with disease resistance in previous reports. * and ** represent *p*-value < 0.05 and *p*-value < 0.01, respectively

To validate the transcriptome sequencing results, 11 DEGs associated with disease resistance, including 5 up-regulated and 6 down-regulated DEGs, were analyzed by qRT-PCR (Fig. 8). Melting curves showed a unique peak for all genes, indicating good primer specificity. Rice *ubiquitin* was used as the internal control to normalize the Ct values. There was a strong correlation (R^2^ = 0.92) between the qRT-PCR and RNA-seq results (Fig. 8a). The expression of *OsWAK91*, *OsGLN2*, *OsRLCK5*, *SDRLK-3,* and *LOC_Os12g28100* was higher in the resistant genotype T49 compared to the susceptible genotype E29, which we used * and ** to represent *p*-value < 0.05 and *p*-value < 0.01, respectively (Fig. 8b). These genes may positively regulate sheath blight resistance. Meanwhile, the expression of *OsMPK17, Xa1*, *Os4CL3*, *OsSERK2*, *Spl7,* and *OsMESL* was higher in E29, suggesting negative regulation (Fig. 8b). These results confirmed the expression trends from the RNA-seq data and the reliability of the Illumina sequencing results.

## Discussion

### Sheath Blight Resistance is Affected by the Level of Ploidy

Autotetraploid rice is derived from its diploid ancestor by colchicine-induced genome duplication (Wu et al. 2021). Compared to its diploid counterpart, autotetraploid rice exhibits greater genetic variation, larger grain size, heavier grain weight, and increased protein content (He et al. 2011; Shahid et al. 2011; Tu et al. 2007). It also shows potential benefits in terms of biomass yield, nutrition, and tolerance to biotic and abiotic stress, which are crucial for climate change adaptation (Chen et al. 1987; Li et al. 2016; Shahid et al. 2012; Song and Zhang 1992). However, the disease resistance of autotetraploid rice has not been documented so far. This study is the first to evaluate the sheath blight field resistance of autotetraploid rice and to examine the effect of chromosome doubling on rice sheath blight resistance. We showed that ploidy is a factor affecting the sheath blight field resistance. Our findings revealed that chromosome doubling enhanced the sheath blight resistance in 45.71% of the autotetraploid genotypes relative to their diploid ancestors, reduced the resistance in 5.71% of the genotypes, and led to unstable resistance in 48.57% of the genotypes across three sowing dates, with some showing increased resistance in certain environments and decreased resistance in others. We further analyzed the average resistance of 35 genotypes. Based on the data, 68.6% of the autotetraploid genotypes showed enhanced resistance, 20% showed lower resistance, and 11.4% showed nearly equal resistance to their diploid counterparts (Additional file 1: Table S15). This suggested that the autotetraploid lines generally showed increased resistance at three different sowing dates.

There are several factors that can affect the evaluation of sheath blight disease resistance. The first factor is plant height. Plant height can significantly impact sheath blight disease resistance, even when both plants have the same lesion length. For example, if both Plant A and Plant B have a lesion length of 50 cm, Plant A is 50 cm tall and Plant B is 100 cm tall, the 50 cm lesion would have a greater impact on Plant A’s panicle and cause yield loss, while Plant B, which is 100 cm tall, would experience less damage from the same 50 cm lesion. Significant negative correlations (*p* < 0.01) between plant height and disease rating were detected in the present study (Additional file 1: Table S19), as found in many previous reports. The reason why negative correlations were often detected between plant height and disease rating has been reviewed by Zeng et al. (2015). The second factor is the heading date. In our experiment conducted in Hangzhou, most rice cultivars headed at around 90 days (during the summer when planted in May each year). At that time, the temperature was very suitable for sheath blight disease pathogens. However, we observed that some cultivars with a heading date of 120 days (headed at autumn) were less susceptible to sheath blight, not because they were more tolerant to the disease, but because the cooler autumn temperatures restricted the growth of the pathogen. The third factor is the thickness of the culm or the lignin content of the shoot cell wall. A higher lignin content in the cell wall can inhibit the growth of the sheath blight pathogen. The fourth factor is the expression level of resistance genes, such as chitinase genes, which can degrade the cell wall of the sheath blight pathogen. In addition to these factors, there are others that can influence the evaluation of sheath blight disease resistance. Given that sheath blight disease resistance can be affected by many factors, studying this disease is challenging. Our objective is to focus on comparing 35 autotetraploid cultivars with their corresponding 35 diploid cultivars. We planted them in the same environment and used the same evaluation method. Lesion length, measured with a ruler, is an objective way to evaluate the damage caused by the pathogen on rice plants. Disease rating, on the other hand, is a subjective way to assess the severity of the damage. As seen from the results, among the 35 genotypes, 17 (48.57%) displayed unstable resistance in the three sowing dates. Based on our past experience, we believe that even when using four or more environments (we used three in this paper), there can still be unstable results. It suggested that sheath blight is complex to study. However, we provide evidence that chromosome doubling, specifically using autotetraploids, offers a new way to increase sheath blight disease resistance.

### More Down-Regulated Genes in Autotetraploid Rice after Inoculation with R. solani

With advances in sequencing technology, RNA-seq has been widely used in research on diploid and autotetraploid plants (Xie et al. 2023). Autotetraploid is an ideal system to study gene dosage effects and has advantages over diploid in biological and economic traits (Martin et al. 2012). Gene expression differences lead to morphological changes between different ploidy cucumbers. Transcriptome analysis of diploid and autotetraploid cucumbers showed more DEGs in autotetraploid, suggesting increased gene activity in polyploid plants (Xie et al. 2023). Other studies revealed fewer DEGs in drought stress response, but more up-regulated DEGs were annotated in autotetraploid versus diploid trifoliate orange (*Poncirus trifolita*) (Wei et al. 2019). Additionally, resistant genotypes detected earlier and stronger transcriptional responses to *R. solani* invasion than susceptible genotypes (Kumari et al. 2017; Samal et al. 2022; Zheng et al. 2018).

In this study, we found that the autotetraploid rice (T49) recruited more down-regulated resistance genes than the diploid cultivar 'Bengal' from which T49 was derived at both 24 hpi and 48 hpi (Table 3). We analyzed these down-regulated genes and found that they participate in the ‘plant-pathogen interaction (ko04626)’, ‘MAPK signaling pathway (ko04016)’, ‘Plant hormone signal transduction (ko04075)’, and other KEGG pathways. In the ko04626 pathway, two genes were down-regulated in diploid and nine genes were down-regulated in autotetraploid at 24 hpi. At 48 hpi, no genes were down-regulated in diploid and ten genes down-regulated in autotetraploid. In the ko04016 pathway, one gene was down-regulated in diploid and five genes were down-regulated in autotetraploid at 24 hpi. At 48 hpi, there were zero genes down-regulated in diploid and six genes down-regulated in autotetraploid. In the ko04075 pathway, there were no genes down-regulated in diploid at 24 hpi, while five genes were down-regulated in autotetraploid. At 48 hpi, there were no genes down-regulated in diploid and four genes down-regulated in autotetraploid. These results indicate that the enhanced sheath blight resistance in autotetraploid rice may be related to the greater number of down-regulated genes participating in the ‘plant-pathogen interaction (ko04626)’, ‘MAPK signaling pathway (ko04016)’, and ‘Plant hormone signal transduction (ko04075)’ pathways.

The diploid rice has two sets of 12 chromosomes (2n = 24), while autotetraploid rice has four sets of 12 chromosomes (4n = 48). It is evident that the presence of four sets of 12 chromosomes in autotetraploid rice would result in greater variation in gene expression compared to the two sets of 12 chromosomes in diploid rice. The doubling of chromosomes in autotetraploid rice causes alterations in the expression of many genes, including both disease resistance genes and other genes unrelated to disease resistance. The alterations in non-disease-related genes may be more extensive in autotetraploid rice than those in disease resistance genes. Therefore, the number of up-regulated genes is much higher in autotetraploid rice compared to the up-regulated genes caused by inoculation (Table 3). This may explain why chromosome doubling results in a greater number of up-regulated genes than inoculation.

### The Ubiquinone/Terpenoid Quinone and Diterpenoid Biosynthesis Pathways May Play Critical Roles in the Response to *R. solani* Infection in Different Ploidy Rice

KEGG Pathway Analysis of the DEGs in transcriptome analysis revealed pathways related to plant-pathogen interaction (KO04626), plant hormone signal transduction (KO04075), MAPK signaling (KO04016), starch/sucrose metabolism (KO00500), phenylpropanoid biosynthesis (KO00940), diterpene biosynthesis (KO00904), and ubiquinone and other terpenoid quinone biosynthesis (KO00130). Similar regulatory pathways have been reported in previous sheath blight studies (Das et al. 2022; Shi et al. 2020; Yang et al. 2023). GO enrichment showed DEGs were associated with defense response, carbohydrate metabolism, ATP binding, and protein kinase activity (Additional file 2: Fig. S4). MapMan analysis indicated the DEGs were mainly involved in signal regulation, pathogenesis-related (PR) proteins, and proteolysis (Additional file 2: Fig. S5). Another study found 5965 DEGs between Lemont and GD66 after *R. solani* infection, with top enrichment in plant–pathogen interactions, plant hormone signal transduction, and MAPK signaling (Liu et al. 2022), which is similar with the present result. Previous studies have shown that photosynthesis, photorespiration, JA signaling, the expression of PR genes, key transcription factors, PAL genes, and other defense-related pathways may contribute to rice sheath blight resistance (Yang et al. 2022; Zhang et al. 2017). Compared to diploids, tetraploid honeysuckle had up-regulated several important genes involved in plant hormone signal transduction and plant pathogen interaction, reflecting enhanced adaptability and resistance of tetraploid species (Wang et al. 2020).

Notably, specific DEG expression patterns in this study were enriched in ubiquinone/terpenoid quinone biosynthesis, phenylpropanoid metabolism, and diterpenoid biosynthesis in different ploidy rice. Shi et al. (2020) found sesquiterpenoid/triterpenoid and phenylpropanoid biosynthesis were significantly up-regulated in the moderately resistant Yanhui-888 versus susceptible Jingang-30 after *R. solani* infection. Secondary metabolites like phenylpropanoids, lignin, waxes, terpenes, and flavonoids play critical roles in sheath blight resistance (Samal et al. 2022; Zheng et al. 2018). Terpene synthase is a key enzyme in terpenoid biosynthesis, and a tolerant genotype up-regulated a terpene synthase gene during the *R. solani-*treated (Karunanithi and Zerbe 2019; Samal et al. 2022). *R. solani*-infected rice plants produced diterpenoid phytoalexins have been reported in earlier studies (Bera and Purkayastha 1999; Schmelz et al. 2014). Here, ubiquinone/terpenoid quinone biosynthesis was positively regulated in autotetraploid rice, while diterpenoid biosynthesis was negatively regulated, indicating complex molecular defense responses to *R. solani* infection between different ploidy rice. The up- and down-regulation of the identified DEGs in this research has been annotated on these two pathway maps (Additional file 2: Fig. S2 and S3). These genes could participate in regulating disease resistance in autotetraploid rice, and their functions need further validation. Overall, this study represents an initial step to elucidate molecular mechanisms involved in *R. solani* resistance. Autotetraploid rice may utilize unique pathways to regulate sheath blight resistance, with different ploidy materials conferring distinct mechanisms. The transcriptome data will help guide further research on novel disease management strategies in rice.

### The Regulation of Defense Related Genes Contributed to Sheath Blight Tolerance in Autotetraploid Rice

With the help of transcriptome technology, studies have reported on plant disease resistance gene regulation to elucidate molecular resistance mechanisms (Gao et al. 2016; Jain et al. 2016). To our knowledge, this is the first study to apply comparative transcriptome analysis to explore gene expression patterns in resistant autotetraploid and susceptible diploid rice against *R. solani.* The identified DEGs may facilitate exploring autotetraploid sheath blight resistance. The most represented DEGs were previously related to blast resistance, followed by bacterial leaf blight, sheath blight, and other defense responses (Fig. 7e). Despite the economic impact, sheath blight is less studied molecularly compared to rice blast and bacterial blight (Molla et al. 2019). Several DEGs implicated in disease resistance were more highly expressed in T49. *OsRLCK5* enhances rice sheath blight resistance by positively regulating reactive oxygen species (ROS) through the ascorbate glutathione system (Wang et al. 2021). *OsGLN2* may confer fungal pathogen defense, while *OsWAK91* positively regulates rice blast fungus resistance (Akiyama et al. 2004; Delteil et al. 2016). Other resistance regulators like *IDD3* (Sun et al. 2020), *pit* (Kawano et al. 2010)*, ASCAB9-A* (Liu et al. 2019)*,* serine/threonine protein kinase receptor precursor (*LOC_Os01g57560*), and NBS-LRR disease resistance protein (*LOC_Os12g28100*) showed higher expression in the resistant T49 than susceptible E29, potentially contributing to autotetraploid rice sheath blight tolerance (Figs. 7e, 8). These results provide insights into genes and pathways involved in sheath blight resistance in autotetraploid rice.

Some DEGs negatively regulating disease resistance were down-regulated in T49 compared to E29 (Fig. 8). *OsMPK17* negatively regulates *Xa21,* which mediates bacterial blight (*Xoo*) resistance in rice (Zhu et al. 2022). *Osmesl* mutant and *OsMESL* RNAi lines show enhanced resistance to bacterial blight, sheath blight and blast (Hu et al. 2021). *OsSERK2* positively mediates bacterial blight resistance through *XA21* (Zuo et al. 2014). *OsSPL7* regulates reactive oxygen species and biotic/abiotic stress responses in rice, and both *SPL7OX* and *spl7ko* enhance resistance to *Magnaporthe oryzae* and blight (Hoang et al. 2019). *Os4CL3* (*LOC_Os02g08100*) inhibits *M. grisea* penetration, enhancing early-stage blast resistance (Li et al. 2020b). Sheath blight is caused by fungal rather than bacterial pathogens, and these bacterial pathogen response genes were down-regulated in T49 compared to E29 (Fig. 8). The differential expression of these genes highlights candidate pathways for exploring sheath blight resistance mechanisms in autotetraploid rice. These results revealed the key pathways and genes involved in the differential sheath blight resistance in different-ploidy rice and provide potential targets for improving rice resistance.

## Conclusions

This work compared the field resistance to sheath blight of 35 autotetraploid and 35 original diploid rice genotypes across three environments. It revealed that inoculation at the booting stage was optimal for evaluating field sheath blight resistance. Overall, the autotetraploids were generally more resistant than diploids, and chromosome doubling improved resistance in some cases depending on the genotype and environment. This study found that autotetraploid rice recruited a stronger induction of DEGs, especially the defense-related genes like *OsWAK91, OsRLCK5* and *OsGLN2*. It also discovered that the ubiquinone/terpenoid quinone biosynthesis and diterpenoid biosynthesis pathways were differentially regulated in response to *R. solani* infection in different ploidy rice.

### Supplementary Information

Additional file 1. Table S1 Information on 35 autotetraploid genotypes and 35 cultivars from which they derived. Table S2 0–9 rating system for evaluating sheath blight disease rating Table S3 The gene-specific primers used for qRT-PCR. Table S4 Correlation coefficients for field resistance of 35 autotetraploid rice genotypes planted on March 1, 2020, July 26, 2020 and February 25, 2021. Table S5 Correlation coefficients for field resistance of 35 diploid rice genotypes planted on March 1, 2020, July 26, 2020 and February 25, 2021. Table S6 Two-way analysis of variance using the lesion length data from 35 autotetraploids and 35 diploids over three cultivation environments. Lesion length was examined at 10 dpi. Table S7 Two-way analysis of variance using the lesion length data from 35 autotetraploids and 35 diploids over three cultivation environments. Lesion length was examined at 28 dpi. Table S8 Two-way analysis of variance using the disease rating data from 35 autotetraploids and 35 diploids over three cultivation environments. Table S9 Three-way analysis of variance using the lesion length data from 35 genotypes over three cultivation environments at 10 dpi. The 35 genotypes consisted of 35 diploids and their corresponding autotetraploids. Table S10 Three-way analysis of variance using the lesion length data from 35 genotypes over three cultivation environments at 28 dpi. The 35 genotypes consisted of 35 diploids and their corresponding autotetraploids. Table S11 Three-way analysis of variance using the disease rating data from 35 genotypes over three cultivation environments. The 35 genotypes consisted of 35 diploids and their corresponding autotetraploids. Table S12 Average lesion length, average disease rating, 7 rating scores for 35 autotetraploid rice genotypes planted in three environments. Table S13 Average lesion length, average disease rating, 7 rating scores for 35 diploid rice genotypes planted in three environments. Table S14 Statistical analysis of 35 diploid and autotetraploid rice genotypes planted in three environments. Table S15 Field resistance statistics and comparison between 35 autotetraploid and diploid rice varieties. Table S16 Average lesion length, average disease rating, 7 rating score for 35 autotetraploid and diploid rice genotypes across three environments. Table S17 Plant height and sheath blight field resistance of the autotetraploid genotypes used for correlation analysis. Table S18 Plant height and sheath blight field resistance of the diploid genotypes used for correlation analysis. Table S19 Correlation coefficients between plant height and sheath blight field resistance for both autotetraploid and diploid genotypes. Table S20 RNA-seq read counts and read mapping statistics for the rice genome. Table S21 The regulation of the DEGs in E29 (E29-0 hpi vs E29-24 hpi). Table S22 The regulation of the DEGs in T49 (T49-0 hpi vs T49-24 hpi). Table S23 The regulation of the DEGs in E29 (E29-0 hpi vs E29-48 hpi). Table S24 The regulation of the DEGs in T49 (T49-0 hpi vs T49-48 hpi). Table S25 Kyoto Encyclopedia of Genes and Genomes (KEGG) pathways for the DEGs in E29 and T49. Table S26 The regulation of the DEGs in ubiquinone and other terpenoid-quinone biosynthesis pathway. Table S27 The regulation of the DEGs in diterpenoid biosynthesis pathway.

Additional file 2. Fig. S1 Significant differences between the variations after inoculation at different growth stages. Fig. S2 The DEGs involved in ubiquinone and other terpenoid quinone biosynthesis pathways. Fig. S3 The DEGs involved in diterpenoid biosynthesis pathways. Fig. S4 The visualization of GO enrichment terms for the total DEGs detected in comparisons between E29 and T49 at three time points. Fig. S5 MapMan analysis of the total DEGs between E29 and T49.

## Data Availability

All data generated or analyzed during this study are included in this article and its supplementary information files.

## Abbreviations

*R. solani*: *Rhizoctonia solani*
DEGs: Differentially expressed genes
SB: Sheath blight
hpi: Hours post inoculation
PDA: Potato dextrose agar
LL: Lesion length
DR: Disease rating
7R: 7-Rating score
dpi: Days post inoculation
E29: Susceptible diploid cultivar Bengal
T49: Resistant autotetraploid cultivar Bengal-4X
FC: Fold change
GSEA: Gene set enrichment analysis
KEGG: Kyoto encyclopedia of genes and genomes
GO: Gene ontology
qRT-PCR: Real-time quantitative PCR
PR: Pathogenesis related
ROS: Reactive oxidative species
JA: Jasmonic acid
MAPK: Mitogen activated protein kinase
