# Supplementary material for: Comparative Field Evaluation and Transcriptome Analysis Reveals that Chromosome Doubling Enhances Sheath Blight Resistance in Rice
Source: Rice (N Y). 2024 Jul 3;17:42. doi: 10.1186/s12284-024-00722-y (PMC11222352; doi:10.1186/s12284-024-00722-y)
Supplement: Supplementary file 2 — Additional file 2. Fig. S1 Significant differences between the variations after inoculation at different growth stages. Fig. S2 The DEGs involved in ubiquinone and other terpenoid quinone biosynthesis pathways. Fig. S3 The DEGs involved in diterpenoid biosynthesis pathways. Fig. S4 The visualization of GO enrichment terms for the total DEGs detected in comparisons between E29 and T49 at three time points. Fig. S5 MapMan analysis of the total DEGs between E29 and T49. [file 12284_2024_722_MOESM2_ESM.docx]

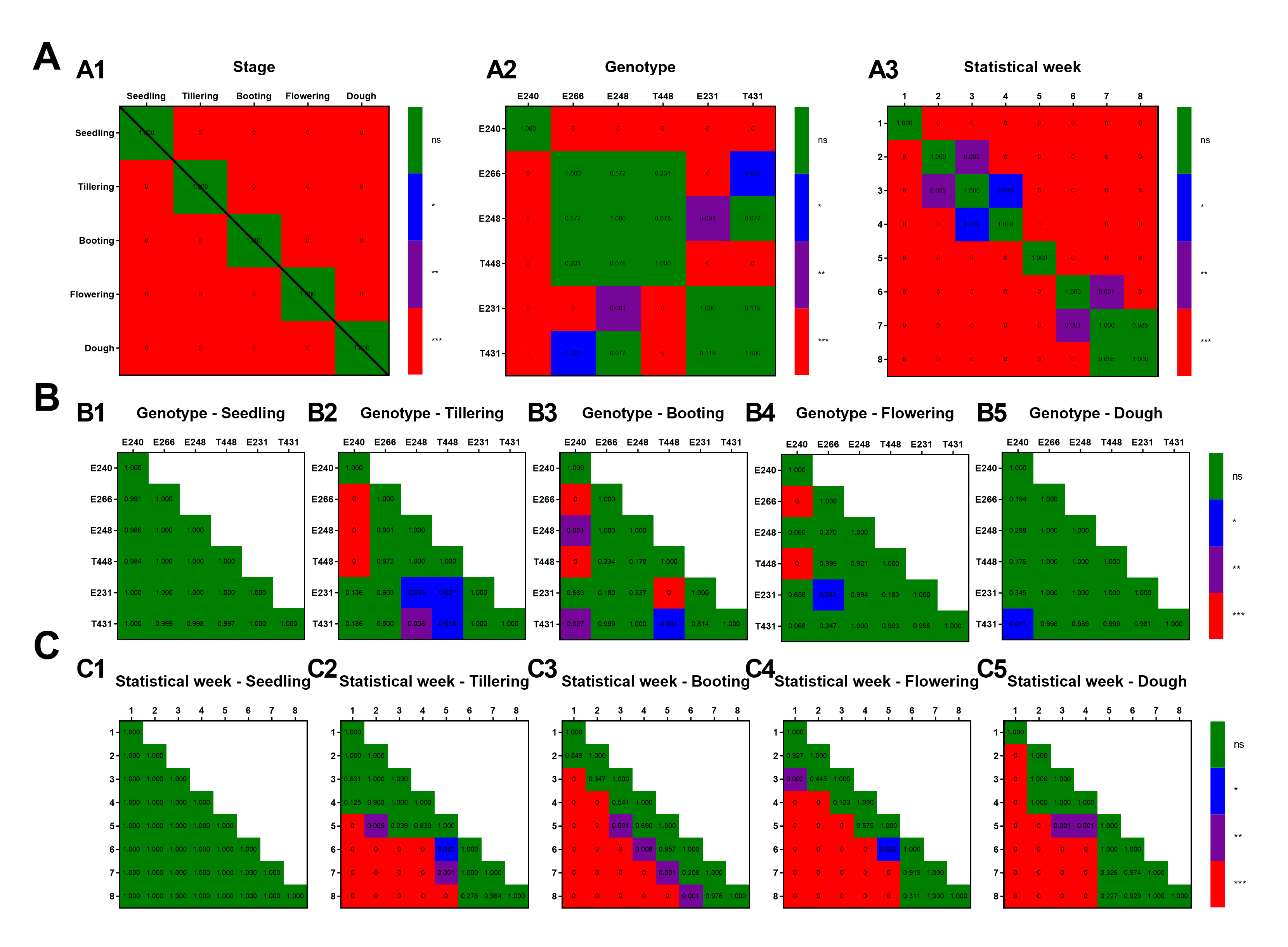


Fig. S1 Significant differences between the variations after inoculation at different growth stages. **A** Significant differences between variations, above and below the diagonal are symmetrical. **B** Significant differences between genotypes inoculated at different stages: B1-B5 indicate inoculation at seedling, tillering, booting, flowering and dough stage, respectively. **C** Significant differences between the lesion length recorded at 8 different weeks (statistical weeks) after inoculation at different stages, and typeset definitions as in B. NS, *, **, *** indicate *p*-value > 0.05, *p*-value < 0.05, *p*-value < 0.01 and *p*-value < 0.001, respectively.


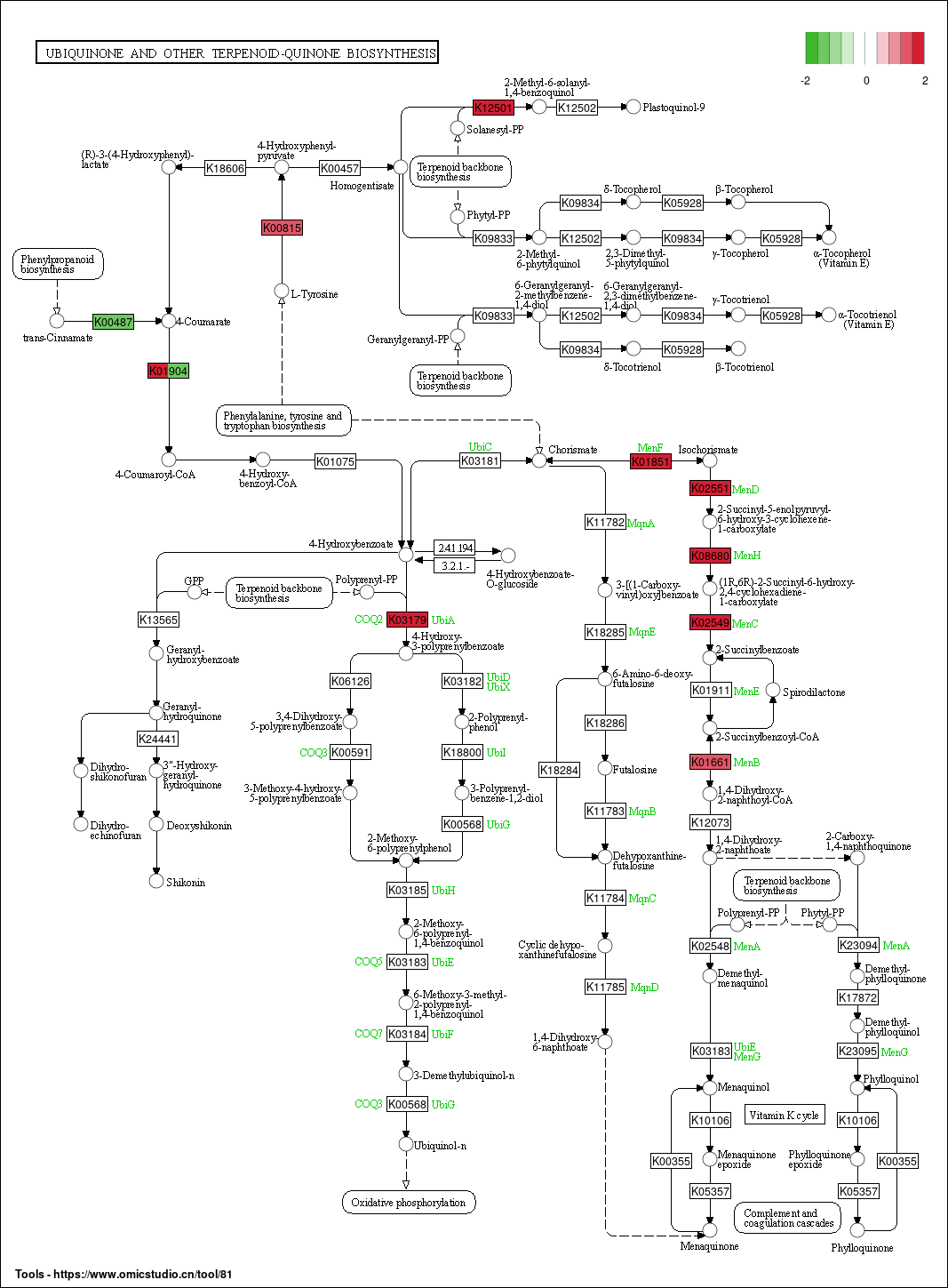


Fig. S2 The DEGs involved in ubiquinone and other terpenoid quinone biosynthesis pathways. Expression values are presented as log_2_FC of genes in T49 compared to E29 at 24 hpi. Red indicates up-regulated genes, and green indicates down-regulated genes.


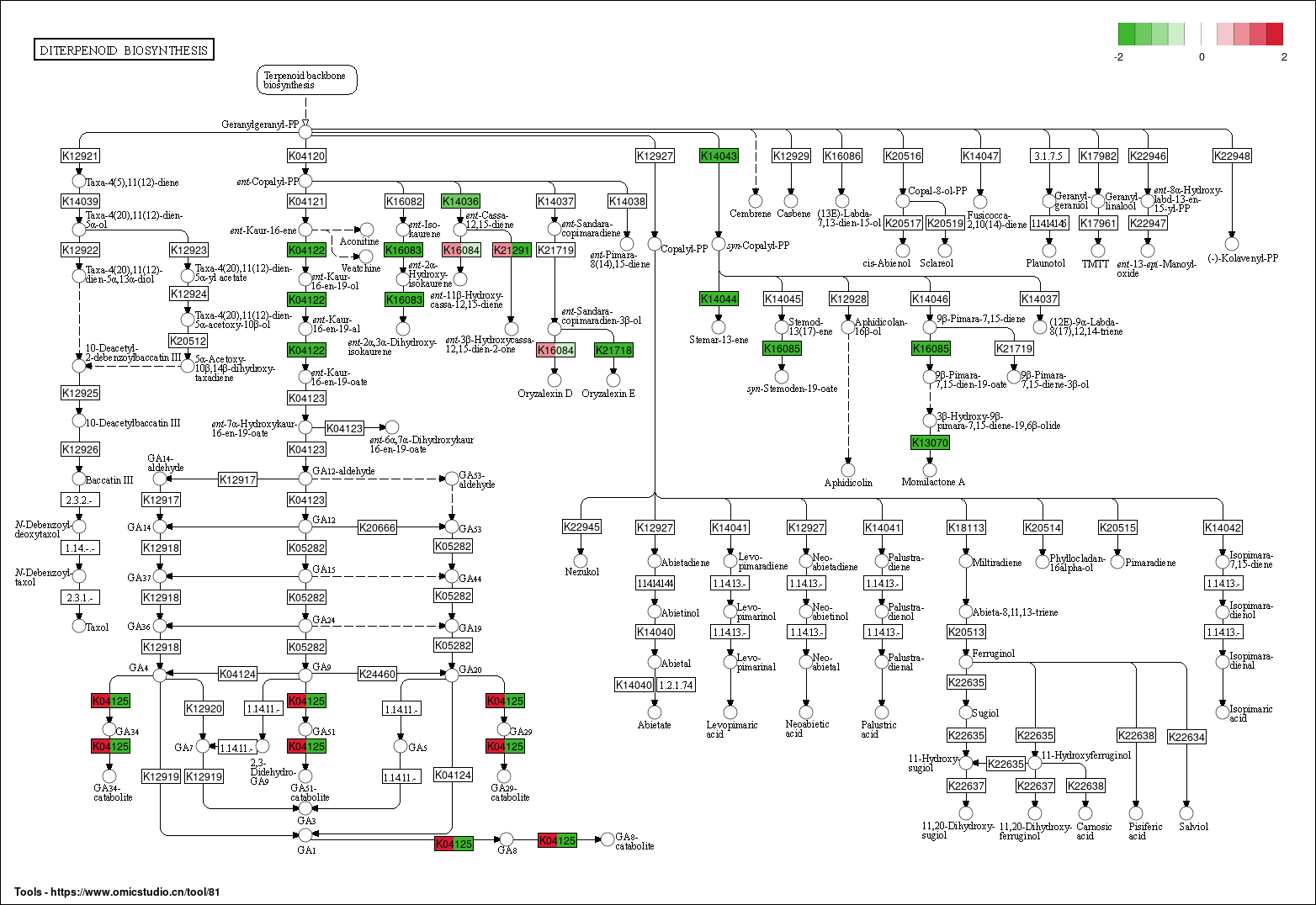


Fig. S3 The DEGs involved in diterpenoid biosynthesis pathways. Expression values are presented as log_2_FC of genes in T49 compared to E29 at 24 hpi. Red indicates up-regulated genes, and green indicates down-regulated genes.


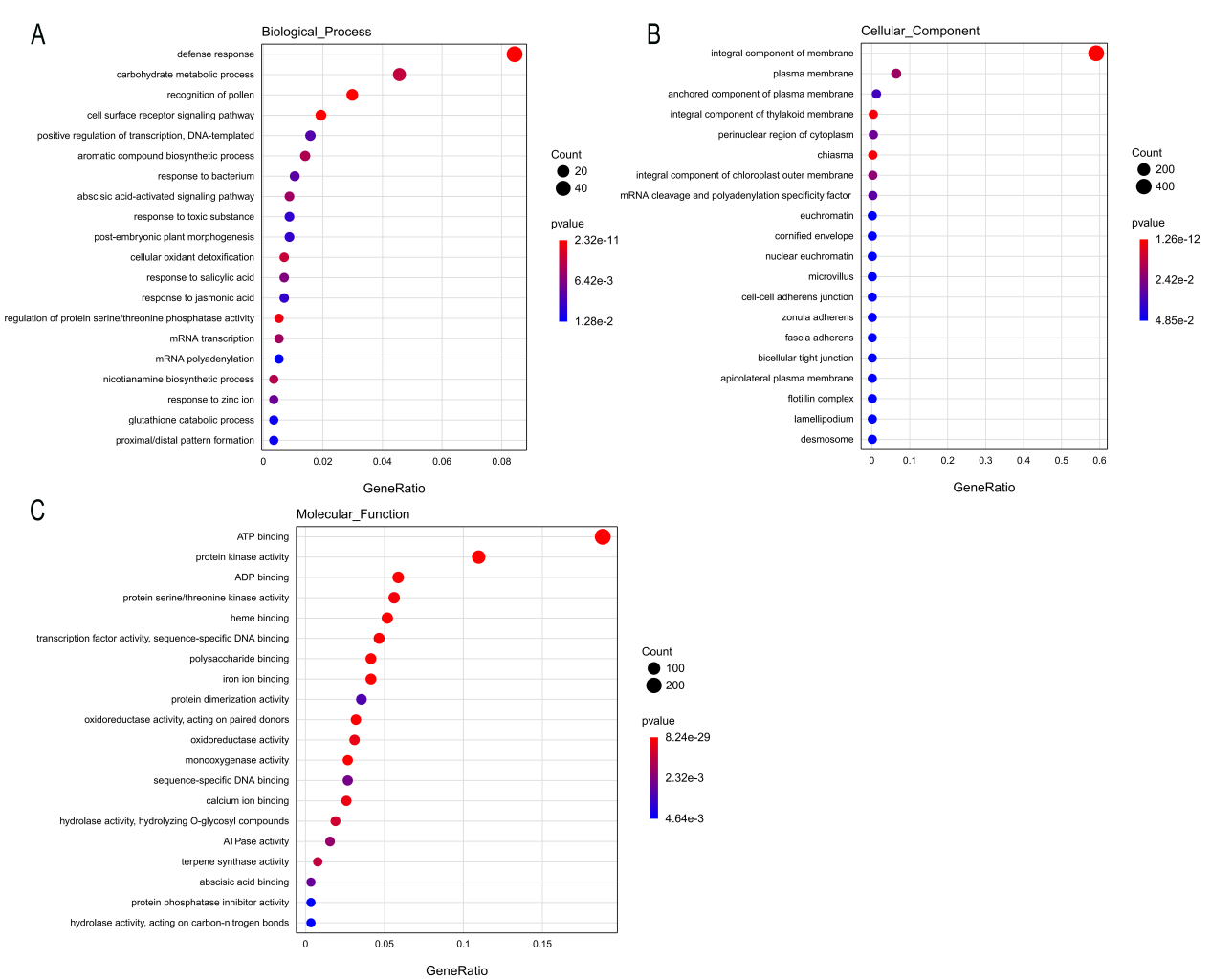
Fig. S4 The visualization of GO enrichment terms for the total DEGs detected in comparisons between E29 and T49 at three time points for Biological_Process (A), Cellular_Component (B) and Molecular Function (C).


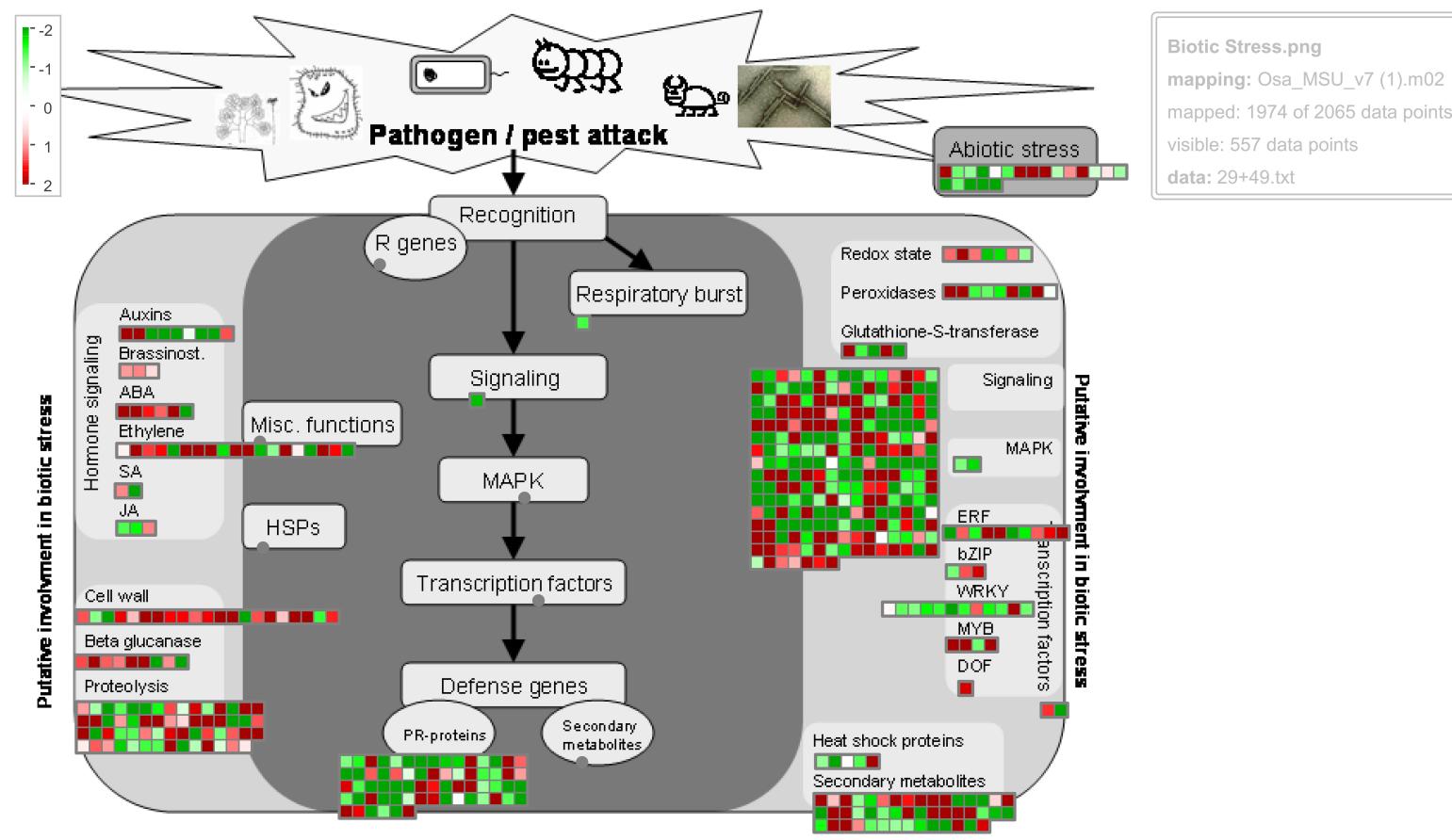


Fig. S5 MapMan analysis of the total DEGs between E29 and T49 based on Log_2_FC values at 24 hpi. Red indicates up-regulated genes, and green indicates down-regulated genes.
